# Xenophagy receptors Optn and p62 and autophagy modulator Dram1 independently promote the zebrafish host defense against *Mycobacterium marinum*

**DOI:** 10.3389/fcimb.2023.1331818

**Published:** 2024-01-09

**Authors:** Jiajun Xie, Annemarie H. Meijer

**Affiliations:** Institute of Biology Leiden, Leiden University, Leiden, Netherlands

**Keywords:** innate immunity, *Mycobacterium*, Dram1, Optn, p62, autophagy, xenophagy, tuberculosis

## Abstract

Anti-bacterial autophagy, also known as xenophagy, is a crucial innate immune process that helps maintain cellular homeostasis by targeting invading microbes. This defense pathway is widely studied in the context of infections with mycobacteria, the causative agents of human tuberculosis and tuberculosis-like disease in animal models. Our previous work in a zebrafish tuberculosis model showed that host defense against *Mycobacterium marinum* (Mm) is impaired by deficiencies in xenophagy receptors, optineurin (Optn) or sequestome 1 (p62), and Damage-regulated autophagy modulator 1 (Dram1). However, the interdependency of these receptors and their interaction with Dram1 remained unknown. In the present study, we used single and double knockout zebrafish lines in combination with overexpression experiments. We show that Optn and p62 can compensate for the loss of each other’s function, as their overexpression restores the infection susceptibility of the mutant phenotypes. Similarly, Dram1 can compensate for deficiencies in Optn and p62, and, vice versa, Optn and p62 compensate for the loss of Dram1, indicating that these xenophagy receptors and Dram1 do not rely on each other for host defense against Mm. In agreement, Dram1 overexpression in *optn/p62* double mutants restored the interaction of autophagosome marker Lc3 with Mm. Finally, *optn/p62* double mutants displayed more severe infection susceptibility than the single mutants. Taken together, these results suggest that Optn and p62 do not function downstream of each other in the anti-mycobacterial xenophagy pathway, and that the Dram1-mediated defense against Mm infection does not rely on specific xenophagy receptors.

## Introduction

Tuberculosis (TB) is one of the most serious infectious diseases worldwide, affecting around 10 million people every year (Chakaya et al., 2022). Despite considerable efforts on controlling the disease, the emergence of antibiotic-resistant mycobacterial strains and limited success in vaccine development continue to pose threats to the world’s health security. This emphasizes the need for exploring novel therapeutic strategies, such as immunotherapy to boost the host immune response against mycobacterial infection (Kilinç et al., 2021; Kiran et al., 2016; Kumar and Kon, 2021). However, insufficient understanding of the early pathogenesis of infection is a limiting factor for the identification of novel therapeutic targets (Bussi and Gutierrez, 2019).

The causative pathogen of TB, *Mycobacterium tuberculosis* (Mtb), belongs to the genus *Mycobacterium*, which contains two more major groups: *M. leprae* and nontuberculous mycobacteria (Jagielski et al., 2016). One of the nontuberculous mycobacteria, *Mycobacterium marinum* (Mm), causes a systemic TB-like disease in cold-blooded vertebrates. Mm is often used as a model organism to study aspects of human TB, due to its close genetic relation with Mtb and the similarities in pathogenesis to the host, including the intracellular survival in macrophages and the induction of tuberculous granuloma formation (Ramakrishnan, 2020; Varela and Meijer, 2022). Mm, like Mtb, contains the region of difference 1 (RD1) virulence locus, encoding for the type VII secretion system ESX-1 and its secreted proteins, ESAT-6 and CFP-10 (Smith et al., 2008). ESAT-6 is known to have membranolytic activity and is required for permeabilizing the membrane of bacteria-containing vesicles, phagosomes, inside infected host cells, thereby disrupting the phagosomal-lysosomal degradation pathway (Gröschel et al., 2016).

By disrupting phagosomal membranes, virulent mycobacteria gain access to the cytosol of their host cell (Gröschel et al., 2016). The primary host mechanism that restricts intracellular replication of cytosolic microbes is autophagy (Deretic et al., 2006; McEwan, 2017; Sharma et al., 2018). Autophagy is a fundamental degradative process delivering cytoplasmic components to the lysosome (Klionsky et al., 2021). This process helps to recycle building blocks by degrading proteins, lipids, and organelles when there is lack of nutrients in cells but also serves as a quality control system that removes misfolded proteins, damaged organelles and infectious organisms (Vargas et al., 2022). While autophagy may occur as a non-selective process (bulk autophagy), different cellular substrates can be captured and degraded in a highly specific manner as well (selective autophagy), which relies on receptor-mediated recognition of the substrate (Farré and Subramani, 2016). Based on the specific type of substrate, different selective autophagy pathways are categorized, such as mitophagy (mitochondria), aggrephagy (misfolded proteins and RNA aggregates), and xenophagy (intracellular pathogens) (Vargas et al., 2022).

Both Mtb and Mm have been shown to be targeted by xenophagy (Gutierrez et al., 2004; Lerena and Colombo, 2011). Once these bacteria invade the cytosol, they become decorated with ubiquitin, which occurs through direct binding to a mycobacterial surface protein or is mediated by the E3 ubiquitin ligase Parkin, a known risk factor for tuberculosis (Manzanillo et al., 2013; Chai et al., 2019). The ubiquitination makes the bacteria substrates for receptor recognition, initiating the xenophagy pathway (Varela et al., 2019; Campos et al., 2022). While xenophagy is an effective means to clear intracellular infections, pathogenic mycobacteria counteract this host defense response using several virulence factors (Shariq et al., 2023).

The receptors mediating xenophagy are known as sequestosome 1-like receptors (SLRs) and include sequestosome-1 (p62/SQSTM1), optineurin (OPTN), nuclear dot protein 52 kDa (NDP52/CALCOCO2), neighbor of BRCA1 gene 1 (NBR1), and TAX1-binding protein 1 (TAX1BP1/CALCOCO3) (Xu et al., 2015). The importance of SLRs in xenophagy is well demonstrated by their role in controlling various invading bacteria, such as Mtb (Franco et al., 2017; Chai et al., 2019), *Shigella* and *Listeria* (Mostowy et al., 2011), *Salmonella typhimurium* (Zheng et al., 2009; Wild et al., 2011; Bansal et al., 2018), and *Staphylococcus aureus* (Gibson et al., 2021). All SLRs share at least two conserved domains: the ubiquitin-binding domain (UBD) and the LC3-interacting region (LIR), a domain that binds to microtubule-associated protein 1A/1B light chain 3B (MAP1LC3/LC3). The receptors recognize the ubiquitinated substrate through the UBD, and the LIR tethers them to the LC3 molecules on autophagosomal membranes. LC3 elongates and sequestrates the substrate inside a double membrane autophagosome that subsequently fuses with lysosomes (Sharma et al., 2018). It has been shown that multiple members of the SLR family can bind to the mycobacterial cell wall (Deretic, 2012; Watson et al., 2012; Chai et al., 2019). However, the contribution of the different SLRs to the host defense and the possible interaction between them is not well understood.

Because xenophagy is an evolutionarily conserved process, we have previously taken advantage of the zebrafish-Mm infection model to study the role of xenophagy and SLRs during mycobacterial infection *in vivo* (Hosseini et al., 2014; Zhang et al., 2019; Muñoz-Sánchez et al., 2020). Using transgenic zebrafish embryos expressing GFP-Lc3 as an autophagy marker, we have shown the presence of Mm in Lc3-labeled vesicles with autophagic morphology (Hosseini et al., 2014; Zhang et al., 2019). Additionally, we demonstrated that Mm is ubiquitinated and that loss-of-function mutation of the SLR genes *optn* or *p62* impairs the autophagic response and thus increases susceptibility to Mm infection in the zebrafish model (Zhang et al., 2019). Moreover, in gain-of-function experiments, wherein *optn* or *p62* were overexpressed by mRNA injection, we observed increased Lc3 colocalization with Mm, as well as increased resistance against Mm infection (Zhang et al., 2019). Thus, these two SLRs play an important role in the innate host defense of zebrafish embryos during mycobacterial infection.

During infection and other stress responses, several mechanisms operate to enhance the activity of autophagic processes. One of the stress-inducible proteins regulating autophagy is DNA damage regulated autophagy modulator 1 (DRAM1). DRAM1 is an evolutionarily conserved protein with six transmembrane domains, localizing mainly to lysosomes but also to autophagosomes, other organelles, and the plasma membrane (Crighton et al., 2006; Mah et al., 2012). Our previous studies of mammalian DRAM1 and its zebrafish homologue Dram1 have demonstrated a protective role against Mm infection in RAW 264.7 mouse macrophages and zebrafish (van der Vaart et al., 2014; Zhang et al., 2020; Banducci-Karp et al., 2023). Dram1 is induced upon recognition of the pathogen through Toll like receptors (TLR) via the myeloid differentiation primary response 88 (Myd88)-nuclear factor-κB (NF-κB) signaling pathway, leading to increased autophagy and lysosomal activity (van der Vaart et al., 2014). While zebrafish with *dram1* knockdown or mutation are hypersusceptible to Mm infection, *dram1* overexpression increases host resistance against Mm (van der Vaart et al., 2014; Zhang et al., 2020). Likewise, knockdown or mutation of *dram1* decreases colocalization of Lc3 and Mm and acidification of Mm-containing compartments, while overexpression increases Lc3 colocalization with Mm (van der Vaart et al., 2014; Zhang et al., 2020). Knockdown of *Dram1* also decreased colocalization of LC3 and Mm in RAW 264.7 macrophages, reduced the acidification of Mm-containing compartments, and impaired the control of infection (Banducci-Karp et al., 2023). Furthermore, DRAM1 colocalizes with Mtb in primary human macrophages (van der Vaart et al., 2014). Together, these studies indicate a conserved role for DRAM1/Dram1 in autophagic defense against mycobacterial infection.

Despite the evidence for the role of xenophagy in defense against mycobacterial infections, the complementarity and functional differences between the different members of the SLR family are poorly understood. In addition, it is not clear whether DRAM1-mediated autophagy enhancement during infection requires SLR activity. Here we took advantage of *optn* and *p62* CRISPR/Cas9-generated knockout zebrafish lines to study the interdependency between these two SLRs and their interaction with the autophagy modulator Dram1. We investigated if Optn and p62 can compensate for each other’s loss-of-function and if Optn/p62 and Dram1 are dependent on each other for restricting Mm proliferation.

Our results revealed that mutation of *optn* can be rescued by *p62* overexpression, and that, vice versa, *optn* overexpression can rescue the *p62* mutant phenotype. Nevertheless, *optn*/*p62* double mutants display a more severe infection phenotype than the single mutants, indicating that these SLRs cannot fully replace each other’s function. The overexpression of *optn* or *p62* could also rescue the infection phenotype of *dram1* mutants, indicating that xenophagy can be enhanced independently of Dram1. However, the host-protective role of Dram1 is clearly demonstrated by the results showing that Dram1 overexpression can rescue the infection phenotype of *optn*/*p62* double mutants, as well as restore Lc3-Mm colocalization in these mutants. These results indicate that this autophagy modulator can activate host-protective autophagic mechanisms even when the functionality of the xenophagy pathway is severely impaired.

## Results

### Selective autophagy receptors Optn and p62 can compensate for each other’s loss-of-function during mycobacterial infection in zebrafish

**Figure 1:**
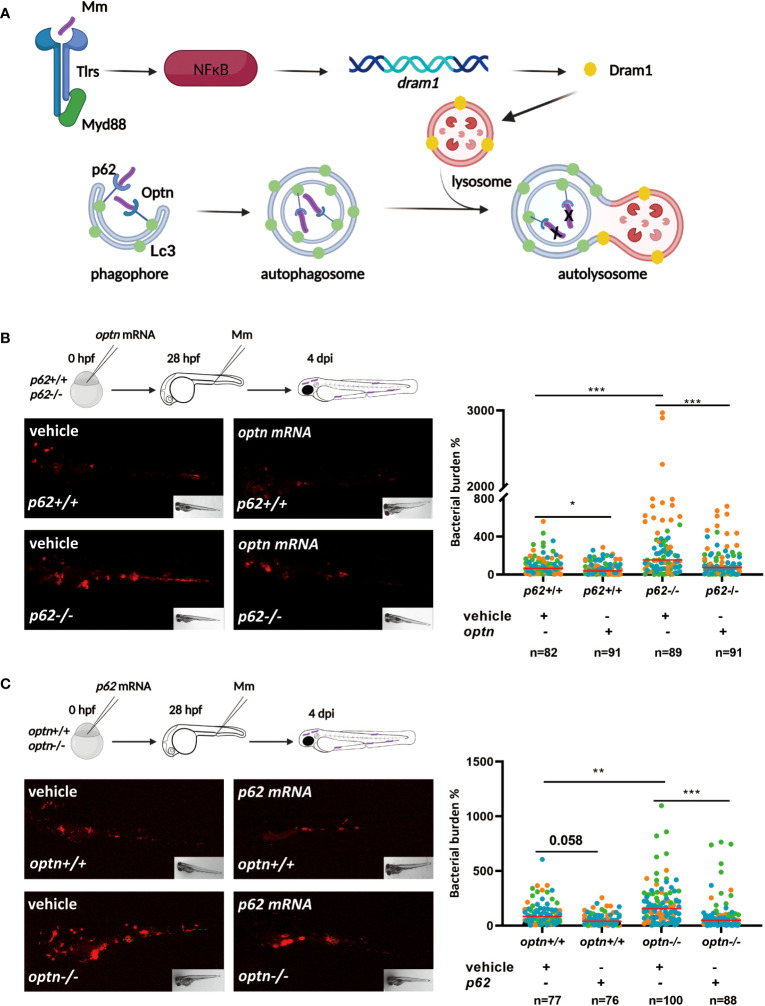
Selective autophagy receptors Optn and p62 can compensate for each other’s loss-of-function during mycobacterial infection in zebrafish. **(A)**. Schematic diagram showing the current model of the roles of autophagy modulator Dram1 and selective autophagy receptors Optn and p62 in defense against Mm infection in zebrafish. Dram1 is induced after pathogen recognition by Tlr-Myd88-NFκB signaling and localizes predominantly to lysosomes, where it is proposed to facilitate the fusion with autophagosomes. The ubiquitin receptors Optn and p62 mediate the selective autophagy (xenophagy) of cytosolic bacteria. Dram1, Optn and p62 have all been shown to contribute to the defense response of the zebrafish host to mycobacterial infection. **(B)** Overexpression of *optn* in *p62* wildtype and mutant background. In the experimental workflow, *optn* mRNA was injected into *p62* +/+ and -/- embryos at the one cell stage, followed by injection of 200 CFU of Mm into the blood island at 28 hpf, and assessment of bacterial burden at 4 dpi (representative images shown). Quantification shows that overexpression of *optn* could decrease bacterial burden independent of *p62*. **(C)** Overexpression of *p62* in *optn* wildtype and mutant background. In the experimental workflow, *p62* mRNA was injected into *optn* +/+ and -/- embryos at the one cell stage, followed by infection and bacterial burden assessment as in **(C)** (representative images shown). Quantification shows that overexpression of *p62* could decrease bacterial burden independent of *optn*. Data are displayed as percentage difference to the control group set at 100% and are accumulated from three independent infection experiments (each data point representing an individual embryo), indicated with different colors. * *p*<0.05, **p<0.01,***p<0.001.

SLRs, such as Optn and p62, are known to recognize bacteria that are ubiquitinated after cytosolic invasion (**Figure 1A**). Previous studies in our lab showed that *optn* and *p62* expression levels are induced during Mm infection. In addition, zebrafish mutants in *optn* or *p62* were found to be hypersusceptible to Mm infection, while overexpression increased resistance of the zebrafish host to Mm infection (Zhang et al., 2019). These similar loss- and gain-of-function effects of both receptors prompted us to further investigate the relationship between them in the zebrafish infection model. First, we studied if overexpression of *optn* or *p62* could compensate for the other receptor’s loss-of function in the response to Mm infection. For these overexpression experiments, mRNAs were synthesized *in vitro* (**Supplementary Figure 1**) and injected into wild type (WT) and *optn* and *p62* mutant embryos at the one-cell stage, using Danieau buffer for mock-injections as control. The embryos were subsequently infected with Mm by injection into the caudal vein at 28 hpf and the bacterial burden was quantified at 4 days post infection (dpi). In agreement with previous results, *optn* and *p62* mutants developed increased bacterial burden compared to the corresponding WT larvae (**Figures 1B, C**) (Zhang et al., 2019). Overexpressions of *optn* and *p62* mRNAs had a reducing effect on the bacterial burden in the WT background, which was in line with previous studies (Zhang et al., 2019), although the effect did not always reach significance (P<0.05 for *optn* mRNA injection and P=0,058 for *p62* mRNA injection) (**Figures 1B, C**). The results of the overexpression studies in the receptor mutants showed that overexpression of *optn* decreased bacterial burden in *p62* mutants with a factor 2.3 compared to the mock-injected controls (**Figure 1B**). Similarly, overexpression of *p62* decreased bacterial burden at 4 dpi in *optn* mutants with a factor 1.9 compared to the mock-injected controls (**Figure 1C**). Taken together, these data confirm that both Optn and p62 contribute to the defense against Mm infection in zebrafish and show that each of these receptors, when overexpressed, can compensate for the loss of the other receptor.

### Double mutation of *optn* and *p62* increases the susceptibility to Mm infection

**Figure 2:**
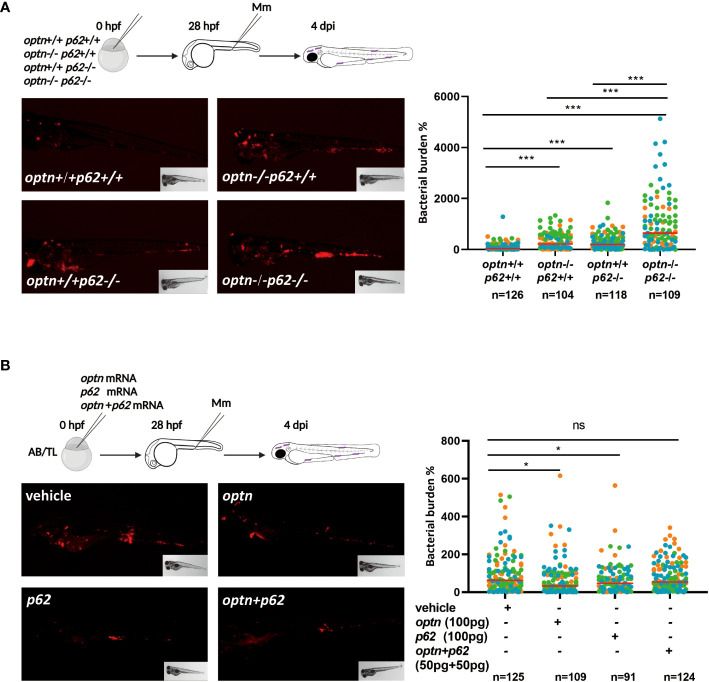
Double mutation of *optn* and *p62* increases the susceptibility to Mm infection but combined overexpression has no additive effect. **(A)** Comparison of single and double *optn*/*p62* mutants. Four different genotypes were infected with 200 CFU of Mm following the indicated experimental workflow and assessed for bacterial burden at 4 dpi (representative images shown). Quantification shows that *optn*/*p62* double mutant embryos were hypersusceptible compared to the wild type and single mutants. **(B)** Comparison of separate and combined overexpression of *optn*/*p62.* One cell stage embryos were injected with 100 pg *optn* mRNA, 100 pg *p62* mRNA, a combination of 50 pg *optn* mRNA and 50 pg of *p62* mRNA, or buffer as a control. The subsequent workflow for infection and analysis of bacterial burden (representative images shown) was the same as in **(A)**. Quantification shows that the combined overexpression of *optn* and *p62* was less effective in reducing bacterial burden. Data are displayed as percentage difference to the control group set at 100% and are accumulated from two **(A)** or three **(B)** independent infection experiments (each data point representing an individual embryo), indicated with different colors. ns, non-significant, * *p*<0.05, ***p<0.001.

Having established that the infection phenotype of *optn* and *p62* receptor mutants can be rescued by overexpressing the other of the two receptors, we investigated if double mutation of *optn* and *p62* would have an additive effect on the susceptibility to Mm infection. Homozygous *optn*/*p62* double mutants were fertile and their offspring did not show any developmental and morphological defects, similarly to the single mutants (Zhang et al., 2019). To compare infection susceptibility between single and double receptor mutants, we injected Mm in *optn*/*p62* double mutants, *optn* single mutants, *p62* single mutants and WT embryos. The infection data showed that *optn*/*p62* double mutant embryos were hypersusceptible compared to the single mutants, resulting in 2.1 and 2.8 fold increased bacterial burden compared to the *optn* and *p62* single mutants, respectively (**Figure 2A**). As a complementary approach, *optn* and *p62* mRNAs were injected into WT embryos, separately and jointly. Separate overexpression of either 100 pg *optn* mRNA or 100 pg *p62* mRNA) decreased bacterial burden by 1.5 and 1.4 fold, respectively. Next, we combined 50 pg *optn* mRNA and 50 pg of *p62* mRNA to reach the same total amount of mRNA. This joint overexpression of *optn* and *p62* did not result in a significant decrease of the bacterial burden (**Figure 2B**). This result, together with the double mutant analysis, supports that Optn and p62 have additive effects on defense against Mm infection, but cannot fully replace each other function.

### Optn and p62 protect against Mm independently of the autophagy modulator Dram1

**Figure 3:**
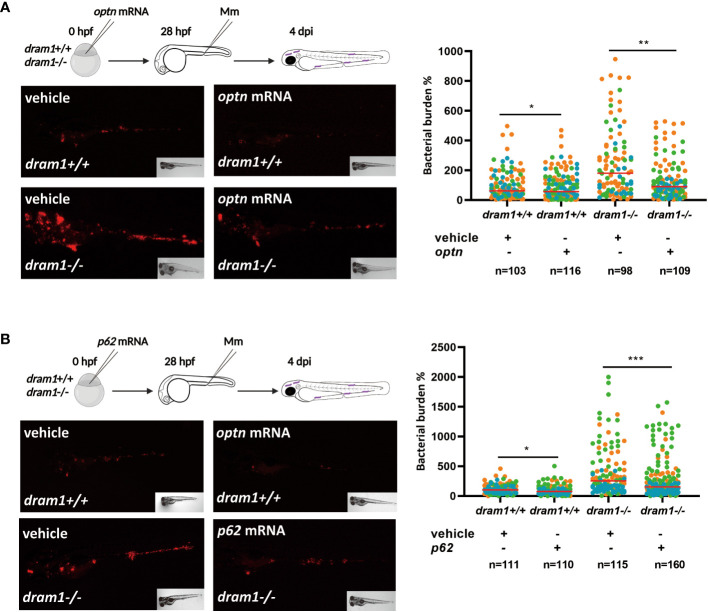
Optn and p62 can protect against Mm independently of the autophagy modulator Dram1. **(A)** Overexpression of *optn* in *dram1* wildtype and mutant background. In the experimental workflow, *optn* mRNA was injected into *dram1* +/+ and -/- embryos at the one cell stage, followed by injection of 200 CFU of Mm into the blood island at 28 hpf and assessment of bacterial burden at 4 dpi (representative images shown). Quantification shows that *optn* overexpression could decrease bacterial burden independent of *dram1*. **(B)** Overexpression of *p62* in *dram1* wildtype and mutant background. Overexpression of *p62* was studied by an experimental workflow analogous to that in **(A)** and found to decrease bacterial burden independent of *dram1.* Data are displayed as percentage difference to the control group set at 100% and are accumulated from three independent infection experiments (each data point representing an individual embryo), indicated with different colors. ns, non-significant, * *p*<0.05, **p<0.01, ***p<0.001.

Dram1 is known as an infection-inducible protein that protects the zebrafish host against Mm infection, similar to Optn and p62 (van der Vaart et al., 2014; Zhang et al., 2019). Dram1 is thought to promote autophagic flux downstream of SLRs but also to initiate autophagosome biogenesis (van der Vaart et al., 2014) (**Figure 1A**). In order to clarify the relationship between the different players in anti-bacterial autophagy, we asked if the SLRs rely on Dram1 for their role in the defense against Mm infection. To answer this question, we injected *optn* mRNA into *dram1* mutant and WT zebrafish embryos and performed Mm infection as described above. Quantification of bacterial burden showed that *optn* overexpression decreased bacterial burden in *dram1* mutants as well in the WT group, with a factor 1.7 and 1.2, respectively (**Figure 3A**). Similarly, we injected *p62* mRNA to *dram1* mutants and the corresponding WT zebrafish embryos, and found that overexpression decreased bacterial burden in both *dram1* mutants and in the WT group, with a factor 1.3 and 1.2, respectively (**Figure 3B**). Taken together, we conclude that the SLRs Optn and p62 promote the defense against Mm infection in zebrafish, even in the absence of the autophagy modulator Dram1.

### Dram1 protects against Mm in the absence of Optn and p62

**Figure 4:**
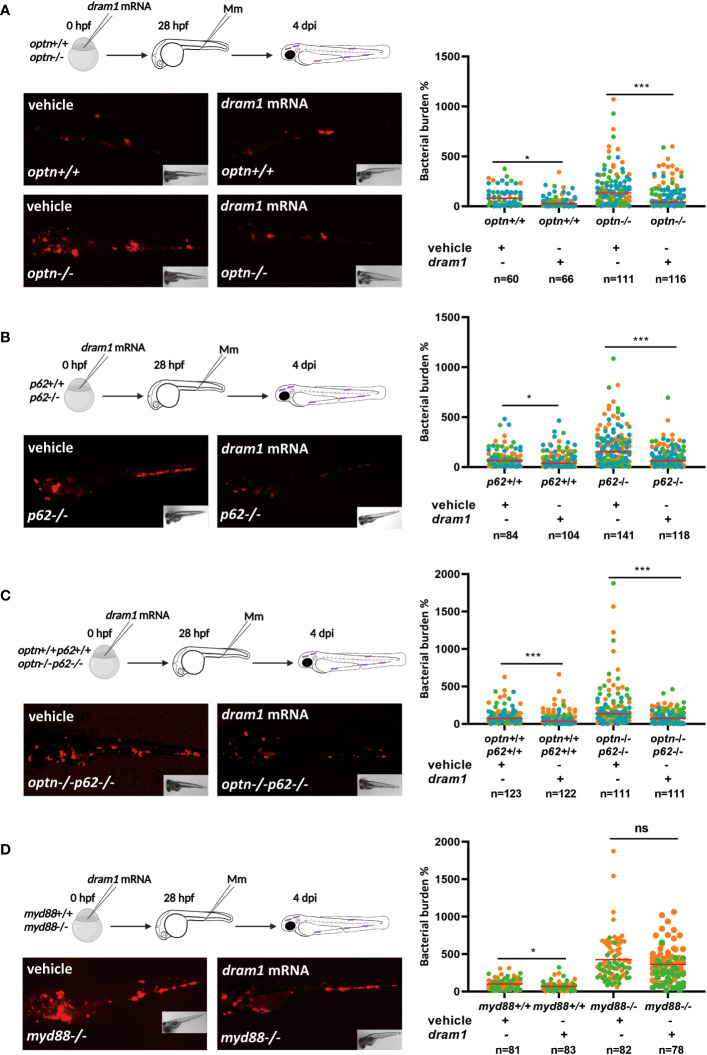
Dram1 can protect against Mm in the absence of Optn and p62. **(A-C)** *dram1* overexpression in single and double mutants of *optn* and *p62*. In the experimental workflow, *dram1* mRNA was injected at the one cell stage into *optn*+/+ and -/- embryos **(A)**, *p62*+/+ and -/- embryos **(B)**, *optn*+/+*p62*+/+ and *optn*-/-*p62*-/- embryos **(C)**, followed by injection of 200 CFU of Mm into the blood island at 28 hpf and assessment of bacterial burden at 4 dpi (representative images shown). Quantifications show that *dram1* could decrease bacterial burden independent of the loss of *optn* **(A)**, *p62* **(B)** or both receptors **(C)**. **(D)** Overexpression of *dram1* in *myd88* wild type and mutant background. *dram1* mRNA was injected into *myd88+/+* and *myd88-/-* embryos at the one cell stage, followed by infection and bacterial burden assessment as in **(A-C)** (representative images shown). Quantification showed that *dram1* overexpression could not decrease bacterial burden in *myd88* mutants. Data are displayed as percentage difference to the control group set at 100% and are accumulated from three **(A-C)** or two **(D)** independent infection experiments (each data point representing an individual embryo), indicated with different colors. ns, non-significant, * *p*<0.05, ***p<0.001.

Since Optn and p62 are able to function in the absence of Dram1, we subsequently investigated if, *vice versa*, Dram1’s function depends on Optn or p62. To this end, *dram1* mRNA was injected to *optn* mutant and WT embryos, followed by Mm injection. The infection data showed that *dram1* overexpression could decrease bacterial burden when *optn* is mutated (1.9 and 1.7 fold decrease in WT and *optn* mutants, respectively) (**Figure 4A**). Similarly, injection of *dram1* mRNA into *p62* mutant and WT embryos showed that *dram1* was also able to decrease bacterial burden in the absence of *p62* (1.3 and 2.0 fold decrease in WT and *p62* mutants, respectively) (**Figure 4B**). Then we examined if this effect of *dram1* overexpression is still seen in *optn* and *p62* double mutant embryos. When we overexpressed *dram1* in *optn*/*p62* double mutants, we observed a 2.3 fold decreased bacterial burden compared to mock-injected controls (**Figure 4C**). These data demonstrate that Dram1 does not require the presence of Optn or p62 or both of these receptors to augment the host defense against Mm infection in the zebrafish model.

As a control for the specificity of the *dram1* overexpression phenotypes, we decided to check the effect of *dram1* overexpression under more general immunodeficient conditions. For this purpose, we used a previously characterized *myd88* mutant zebrafish line (van der Vaart et al., 2013). Myd88 functions as an adaptor molecule in TLR and Interleukin 1 receptor signaling (**Figure 1A**). This protein is known to play a central role in the activation of the innate immune response and the *myd88* mutation results in hypersusceptibility of the zebrafish host to Mm (van der Vaart et al., 2013). After injection of *dram1* mRNA in *myd88* mutant embryos, there was no significant effect on the bacterial burden of this injection in comparison to the mock-injected controls (**Figure 4D**). Thus, although Dram1 enhances the immune response to Mm infection in the absence of Optn or p62, it cannot compensate for general immunodeficiency caused by disruption of Myd88-dependent innate immune signaling.

### Dram1 increases the colocalization between Lc3 and Mm, and acidification of Mm in *optn/p62* double mutant lines

**Figure 5:**
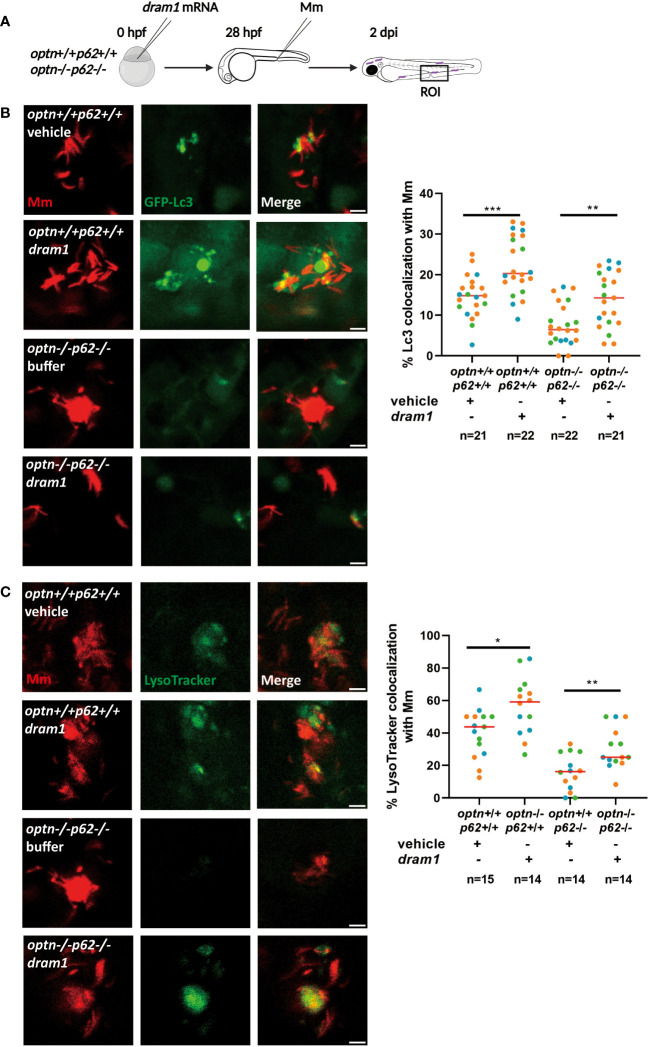
Dram1 increases the colocalization between Lc3 and Mm in *optn/p62* double mutant lines. Effect of *dram1* overexpression on the colocalization between Mm and GFP-Lc3 in *optn/p62* double mutant and wildtype background. In the experimental workflow **(A)**, *dram1* mRNA was injected into *optn*+/+*p62*+/+ and *optn*-/-*p62*-/- embryos at the one cell stage, followed by injection of 200 CFU of Mm into the blood island at 28 hpf and imaging of a region of interest (ROI) in the caudal hematopoietic region at 2 dpi (representative confocal microscopy images shown). **(B)** Quantification shows that *dram1* overexpression increased Mm/GFP-Lc3 colocalization independent of *optn* and *p62*. Complete or partial overlap of GFP-Lc3 with Mm was considered as co-localizing events. Data are displayed as percentage of GFP-Lc3-positive Mm clusters relative to the total number of Mm clusters and are accumulated from three independent infection experiments, of which the data points (each representing a single ROI from an individual embryo) are indicated with different colors. **(C)** Quantification shows that *dram1* overexpression increased Mm and LysoTracker colocalization independent of *optn* and *p62*. Complete or partial overlap of LysoTracker with Mm was considered as co-localizing events. Data are accumulated from three independent experiments (each data point representing a single ROI from an individual embryo)* *p*<0.05, ** *p*<0.01.

From our previous work, we know that deficiency of Optn or p62 affects the colocalization of Mm with the fluorescent autophagy marker GFP-Lc3 (Zhang et al., 2019). Here, we investigated if GFP-Lc3 can still be recruited to Mm when both *optn* and *p62* are mutated. We injected Mm into GFP-Lc3-expressing *optn*/*p62* double mutant embryos and observed that GFP-Lc3 punctae were still formed colocalizing with Mm, although at a lower frequency than in the corresponding WT group (**Figure 5**). We hypothesized that, as a compensatory mechanism, other selective autophagy receptors might be expressed at higher levels in the *optn*/*p62* double mutants. To test this hypothesis, we collected samples from infected *optn*/*p62* double mutant embryos and analyzed the expression levels of *ndp52* and *nbr1 by* qPCR. While *nbr1* expression showed no significant difference, the *ndp52* mRNA level was 2.4 fold higher than in WT control embryos, suggesting that this receptor might (partially) compensate for the loss of *optn* and *p62* (**Supplementary Figure 2**). In addition, because overexpression of *dram1* is known to increase colocalization between GFP-Lc3 and Mm (van der Vaart et al., 2014), we investigated if this phenotype is still found in *optn*/*p62* double mutant embryos. The data showed that overexpressing *dram1* increased the colocalization between GFP-Lc3 and Mm in both WT and *optn*/*p62* double mutant embryos by a factor 1.5 and 2.4, respectively (**Figure 5**).

We then wanted to know if Dram1 is able to promote acidification of Mm-containing vesicles in the absence of both Optn and p62. To answer this question, LysoTracker, a dye that stains lysosomes and other acidic vesicles, was used to stain the Mm-injected embryos. We found that Dram1 increased the colocalization of LysoTracker with Mm in both WT and *optn*/*p62* double mutant zebrafish embryos. In conclusion, Dram1 protects against Mm infection by promoting the interaction of Lc3 with Mm and the acidification of Mm-containing vesicles, even in the absence of both Optn and p62.

## Discussion

While xenophagy has emerged as a central defense mechanism against *Mycobacterium* and other intracellular pathogens, the functional specialization or possible interdependence of components of the pathway remains to be fully elucidated (Shariq et al., 2023). In this study we utilized different zebrafish mutant lines and the well-established zebrafish tuberculosis model to study the interaction of autophagy receptors of the SLR family, Optn and p62, and the autophagy modulator Dram1 in the host defense against mycobacterial infection. This work extends our previous findings that Optn, p62 and Dram1 are important for resistance to Mm infection and confirms that their endogenous expression levels are not sufficient to provide full host resistance (van der Vaart et al., 2014; Zhang et al., 2020). The results indicate a remarkable resilience in the innate immune defense of the zebrafish, demonstrating that deficiencies in either the SLRs or in Dram1 can be compensated for, as overexpression of any of these factors in other mutant backgrounds improved the ability of the zebrafish host to restrict Mm proliferation.

The SLR family members p62, OPTN, NDP52, NBR1 and TAX1BP1 have all been implicated in xenophagy of Mtb (Watson et al., 2012; Chai et al., 2019). Four of them (p62, OPTN, NDP52 and NBR1) were shown to interact with the Mtb surface protein Rv1468c, which mediates xenophagy through its ubiquitin-binding properties (Chai et al., 2019). Deletion of p62 was sufficient to block the Rv1468c-dependent xenophagy of Mtb in RAW 264.7 macrophages, suggesting that p62 and the other SLRs perform non-redundant functions in the anti-mycobacterial defense response (Chai et al., 2019). In agreement, knockdown of either p62 or NDP52 reduced LC3 colocalization with Mtb in RAW 264.7 macrophages. Our results of Mm infection in the zebrafish model are consistent with these findings in that deficiency in either Optn or p62 reduced Lc3 recruitment and resulted in higher bacterial burden (Zhang et al., 2019). However, using the *optn*/*p62* double mutant zebrafish line, we revealed additive effects of Optn and p62 in controlling bacterial burden, indicating that these two SLRs do not have an epistatic relationship in the anti-mycobacterial xenophagy pathway. Furthermore, we observed that overexpression of either Optn or p62 could compensate for the loss of the other SLR, confirming that Optn and p62 do not rely on each other to defend against Mm infection in zebrafish.

The interaction between different SLRs has also been studied in the context of protein degradation and *Salmonella* infection. Similar to our finding, NBR1, another SLR, functions independently of p62 in the autophagosomal clearance of ubiquitinated protein aggregates, even though NBR1 and p62 can physically interact with each other (Kirkin et al., 2009). However, double silencing of p62 and NDP52 had no additive effect in terms of colocalization of LC3 and *S. typhimurium* in HeLa cells and it impaired the antibacterial response similarly as separately silencing of each of two SLRs (Cemma et al., 2011). Thus, it was concluded that p62 and NDP52 function cooperatively in the same pathway. Furthermore, these two receptors were shown to bind to distinct microdomains on the *Salmonella* surface (Cemma et al., 2011; Wild et al., 2011).

OPTN was shown to colocalize to the same microdomains as NDP52, but to different domains as p62 (Wild et al., 2011). Double knockdown of OPTN with either NDP52 or p62 in HeLa cells showed no additive effect on *Salmonella* proliferation in HeLa cells nor on *Salmonella* colocalization with LC3, suggesting OPTN and NDP52 or p62 are interdependent on each other in the anti-*Salmonella* xenophagy pathway (Wild et al., 2011). In contrast, our results have revealed that Optn and p62 can mediate protection against *Mycobacterium* in zebrafish independently from each other. The difference in interaction between Optn and p62 could be a pathogen-specific response or could be related to the higher complexity of the *in vivo* context in our study, where depletion or overexpression of SLRs may also impact on other pathways involved in the control of bacterial proliferation, such as inflammation. Indeed, both OPTN and p62 have been shown to interact with proteins in inflammatory signaling pathways (Oakes et al., 2017). Therefore, the role of SLRs and the interaction between them may depend both on the autophagic response to different pathogens and on the host environment.

DRAM1 has previously been found to regulate the localization of p62 to autophagosomes (Galavotti et al., 2013). In addition, our previous data showed that autophagosome formation due to *dram1* overexpression in Mm-infected zebrafish embryos is blocked by knockdown of *p62* (van der Vaart et al., 2014). However, in our current study, we found that overexpression of Dram1 in single or double mutants of Optn and p62 still resulted in increased colocalization between Lc3 and Mm, in increased acidification of Mm, and in decreased proliferation of Mm. This suggests that Dram1 interacts with compensatory pathways, a hypothesis supported by elevated expression levels of *ndp52* and *nbr1* in *optn*/*p62* double mutants. Another possibility is that Dram1 promotes defense against Mm through the autophagy-related Lc3-associated phagocytosis pathway, as previously shown for *Salmonella* Typhimurium (Masud et al., 2021). Considering that Dram1 is a downstream target of the Tlr-Myd88-NF-κB signaling pathway, we also investigated if Dram1 could rescue the infection susceptibility of *myd88* mutants. These mutants are not only impaired in the autophagy response to Mm, but also display strongly decreased expression levels of innate immune genes and accelerated formation of granuloma-like aggregates in zebrafish (van der Vaart et al., 2013). Our data showed that overexpression of Dram1 did not decrease the Mm infection burden in *myd88* mutants. Therefore, we conclude that Dram1 can compensate for (combined) deficiencies in the xenophagy pathway but not for general immunodeficiency.

There are several possible ways in which DRAM1 may augment the activity of the xenophagy pathway and cooperate with the function of SLRs. First, this could be mediated through effects on autophagy initiation and autophagosome formation. Besides acting as a bridge for linking ubiquitinated substrates and nascent phagophores to form autophagosomes, both OPTN and p62 have also been shown to act as scaffolds to build protein-protein interactions, among others in the ULK1 complex (Moscat et al., 2007; Rui et al., 2015; Yamano and Youle, 2020). The ULK1 complex interacts with ATG13 to increase ULK1 kinase activity and stability, which leads to autophagy initiation, as well as phosphorylation of p62, enhancing its binding affinity to ubiquitin substrates (Katsuragi et al., 2015). DRAM1 is found to enhance ULK1-ATG13 interaction in a dose-dependent manner, thus facilitating the biogenesis of autophagosomes (Lu et al., 2019). In agreement, overexpression of Dram1 in zebrafish was found to increase the number GFP-Lc3 punctae, even in the absence of infection, suggesting that more autophagosomes were formed under these conditions (van der Vaart et al., 2014). A second way in which DRAM1 could augment xenophagy is by promoting the fusion between autophagosomes and lysosomes, which is in line with the increased acidification of Mm that we observed in response to *dram1* overexpression. This function is corroborated by studies in various cell types (Qi et al., 2015; Wu et al., 2018; Geng et al., 2020), and a recent study from our group in RAW 264.7 macrophages supports that DRAM1 promotes the delivery of Mm to lysosomes (Banducci-Karp et al., 2023).

The results of the present study show that Dram1 overexpression rescues the infection susceptibility of *optn*/*p62* double mutant zebrafish, and that, *vice versa*, Optn and p62 could also rescue the infection susceptibility of *dram1* mutants. First, these results indicate the presence of compensatory mechanisms in anti-mycobacterial xenophagy. Second, these results point out that Optn, p62, and Dram1 levels are all limiting factors for the proper control of Mm infection in the zebrafish model. An explanation for the rate-limiting functions of these proteins could be that the autophagic defenses of the host are in competition with counteractive virulence strategies of the pathogen (Shariq et al., 2023). Our results therefore encourage further exploration of autophagy boosting immunotherapies for the treatment of mycobacterial infections, for which a number of drug candidates have been proposed in recent years (Gupta et al., 2016; Paik et al., 2019; Kilinç et al., 2021; Wallis et al., 2021).

## Materials and methods

### Zebrafish culture and lines

Zebrafish lines used in this study were: AB/TL (a cross between the wildtype strains AB and Tuebingen Longfin), *Tg(CMV : GFP-Lc3); dram1^+/+^*, *Tg(CMV : GFP-Lc3); dram1^ibl53/ibl53^*, *Tg(CMV : GFP-Lc3); optn*^+/+^, *Tg(CMV : GFP-Lc3); optn^ibl51/ibl51^*, *Tg(CMV : GFP-Lc3); p62^+/+^*, *Tg(CMV : GFP-Lc3); p62^ibl52/ibl52^, Tg(CMV : GFP-Lc3)* (Zhang et al., 2019; Zhang et al., 2020), and double mutants of the *optn* and *p62* alleles. Genotyping was performed as described in (Zhang et al., 2019) using the following primers: *dram1* forward: AGTGAACGTCCGTGTCTTTCTT; *dram1* reverse: ACATCTTGTCGATACAAAGCGA; *optn* forward: AGTTTAGAGGAGACCCTCCAGC; *optn* reverse: AGAGGTCAGATTCTTCGCATTC; *p62* forward: CATCTTGGATTCATCATTACGTA; *p62* reverse: TCATATGGGGGGTCCTCCT. Zebrafish were maintained according to standard protocols (zfin.org) and in compliance with local animal welfare regulations, as overseen by the Animal Welfare Body of Leiden University (License number: 10612). All embryos were kept in egg water (60 µg/ml Instant Ocean sea salts) at 28.5°C. Embryos were treated with 0.02% ethyl 3-aminobenzoate methanesulfonate (Tricaine, Sigma-Aldrich) for anesthesia before bacterial injections, imaging and fixation.

### Preparation and injection of mRNA

Three pCS2+ expression plasmids containing *dram1*, *optn* and *p62* cDNA used for mRNA synthesis were previously generated and their use for overexpression was validated by qPCR and Western blot analysis (Zhang et al., 2019; Zhang et al., 2020). Plasmids were transformed to *E.coli* cells for amplification. Plasmid extractions were performed by the GenElute plasmid miniprep kit, according to the protocol provided (PLN-70; Sigma-Aldrich). The plasmids were then linearized by enzyme digestions: BamH1 for *dram1*, Not1 for *optn*, and BstB1 for *p62*. The linearized plasmids were used for mRNA synthesis *in vitro* by using the T7 (AM1344, Thermo Fisher), SP6 (AM1340, Thermo Fisher) or T3 (AM1348, Thermo Fisher) mMessage mMachine kits according to the manufacturer’s instructions. Poly(A) tails were then added to the synthesized mRNAs using the Poly(A) Tailing Kit (AM1350, Thermo Fisher). Polyadenylated mRNAs were then purified with lithium chloride. Subsequently, 100 pg mRNA in 1 nl of Danieau buffer (58mM NaCl, 0.7mM KCl, 0.4mM MgSO4, 0.6mM Ca(NO3)2, 5.0mM N-2-hydroxyethylpiperazine-N′-2-ethanesulfonic acid; pH 7.6) was microinjected into one-cell stage embryos.

### Mm culture

The day before embryo infection, Mm M-strain, fluorescently labeled with mWasabi or mCherry, a gift from Kevin Takaki (Department of Microbiology, University of Washington, USA), was cultured in Difco Middlebrook 7H9 medium (Becton Dickinson, BD271310) with 10% BBL Middlebrook albumin-dextrose-catalase (Becton Dickinson, 211887) and 50 µg/ml hygromycin (Sigma-Aldrich, SC-506168A) at 28.5°C in a static incubator. On the day of infection, Mm was washed twice with PBS and optical density (OD) was measured at 600nm. An OD_600nm_ of 1 is equal to 10^8^ CFU/mL (Benard et al., 2012).

### Infection and bacterial burden quantification

Mm bacteria were microinjected into the blood island of embryos at 28 hpf. The injection dose was 200 CFU for all experiments. Embryos were manually dechorionated by tweezers before the injection. Infected embryos were imaged using a Leica MZ16FA stereo fluorescence microscope equipped with a DFC420C color camera, and the number of bacterial pixels per infected fish were obtained from the individual embryo stereo fluorescence images using QuantiFish (http://doi.org/10.5281/zenodo.1182791).

### LysoTracker staining

Mm-infected embryos were immersed in egg water enriched with 10 µM LysoTracker Red DND-99 (L7528, ThermoFisher) for 1h. Larvae were then washed with egg water 3 times before imaging.

### Confocal laser scanning microscopy and image quantification

Mm-infected embryos were anesthetized at 2 dpi and mounted in 1% low melting agarose (140727, SERVA) for imaging using a Leica TCS SP8 confocal microscope with a 40x objective (NA 0.8). For quantification of the colocalization between GFP-Lc3 signal and Mm bacteria, a region in the caudal hematopoietic tissue (CHT) was imaged and GFP-Lc3-Mm colocalization was analyzed by visual stack-by-stack inspection of the confocal Z-stack images.

### qPCR analysis

Embryos were injected with *dram1*, *optn*, and *p62* mRNAs at the one-cell stage as described above. Samples (pools of 15 embryos) were collected every day after mRNA injection and dissolved in TRIzol (Invitrogen, 15596018). After extraction by chloroform, samples were centrifuged at 12,000g for 15 min at 4°C, and the aqueous phase containing the mRNA was transferred to a new tube. 2-propanol was added to the tubes and mixed with the aqueous phase thoroughly to precipitate the mRNA. After washing with 70% ethanol, 1000 ng of each RNA sample, measured with a Nanodrop spectrophotometer (Thermofisher), was converted to cDNA with the C1000 Touch Thermal Cycler using the iScript cDNA Synthesis Kit according to the manufacturer’s protocol (Bio-Rad, 1708890). Samples were then subjected to quantitative PCR (qPCR) using the CFX96 Real-Time System (Bio-Rad) with the iTaq™ Universal SYBR^®^ Green Supermix (Bio-Rad, 1725271). The program was set up as follows: initial denaturation at 95°C for 3 min, then 40 cycles of 95°C for 15 seconds (denaturation), and 60°C for 30 seconds (annealing and extension). A melting curve was included to test for PCR product purity: 55-95°C in 0.5°C increments. Primers were as follows: *dram1* forward: GTGCCCCCTACTCTGAACA; *dram1* reverse: GCGGTATCCGATCACACTCT; *optn* forward: GACTGAACACTATGGCGTGGA; *optn* reverse: GAATGCGAATCTGACCTCT; *p62* forward: GTCATATGGGTCCATCTCCAAT; *p62* reverse: AGGTGGGGCACAAGTCATAA. *tbp* was used as the housekeeping gene: forward: CCTGCCCATTTTCAGTC, reverse: TGTTGTTGCCTCTGTTGCTC.

### Statistical analyzes

Statistical analyzes were performed using R statistical software. Bacterial burden values were normalized and log-transformed to ensure within-group normality, and analyzed downstream by one-way ANOVA. Pairwise comparison with Tukey correction was used to analyze statistical differences between groups. Lc3-Mm colocalization values were analyzed by one-way ANOVA. The qPCR data were analyzed by unpaired t test (ns, non-significance, *p<0.05; **p<0.01; ***p<0.001). All graphs were made in GraphPad Prism8 (mean ± SD is shown).

## Data availability statement

The original contributions presented in the study are included in the article/**Supplementary Material**. Further inquiries can be directed to the corresponding author.

## Ethics statement

The animal study was approved by Dierexperimentencommissie (DEC) Universiteit Leiden. The study was conducted in accordance with the local legislation and institutional requirements.

## Author contributions

JX: Conceptualization, Formal Analysis, Investigation, Visualization, Writing – original draft. AM: Conceptualization, Methodology, Resources, Supervision, Writing – review & editing.
