# Supplementary material for: Xenophagy receptors Optn and p62 and autophagy modulator Dram1 independently promote the zebrafish host defense against Mycobacterium marinum
Source: Front Cell Infect Microbiol. 2024 Jan 9;13:1331818. doi: 10.3389/fcimb.2023.1331818 (PMC10803470; doi:10.3389/fcimb.2023.1331818)
Supplement: Supplementary file 1 [file DataSheet_1.pdf]

Supplementary Material

Xenophagy receptors Optn and p62 and autophagy modulator Dram1 independently promote the zebrafish host defense against *Mycobacterium marinum*

Jiajun Xie and Annemarie H. Meijer*

* author for correspondence: a.h.meijer@biology.leidenuniv.nl

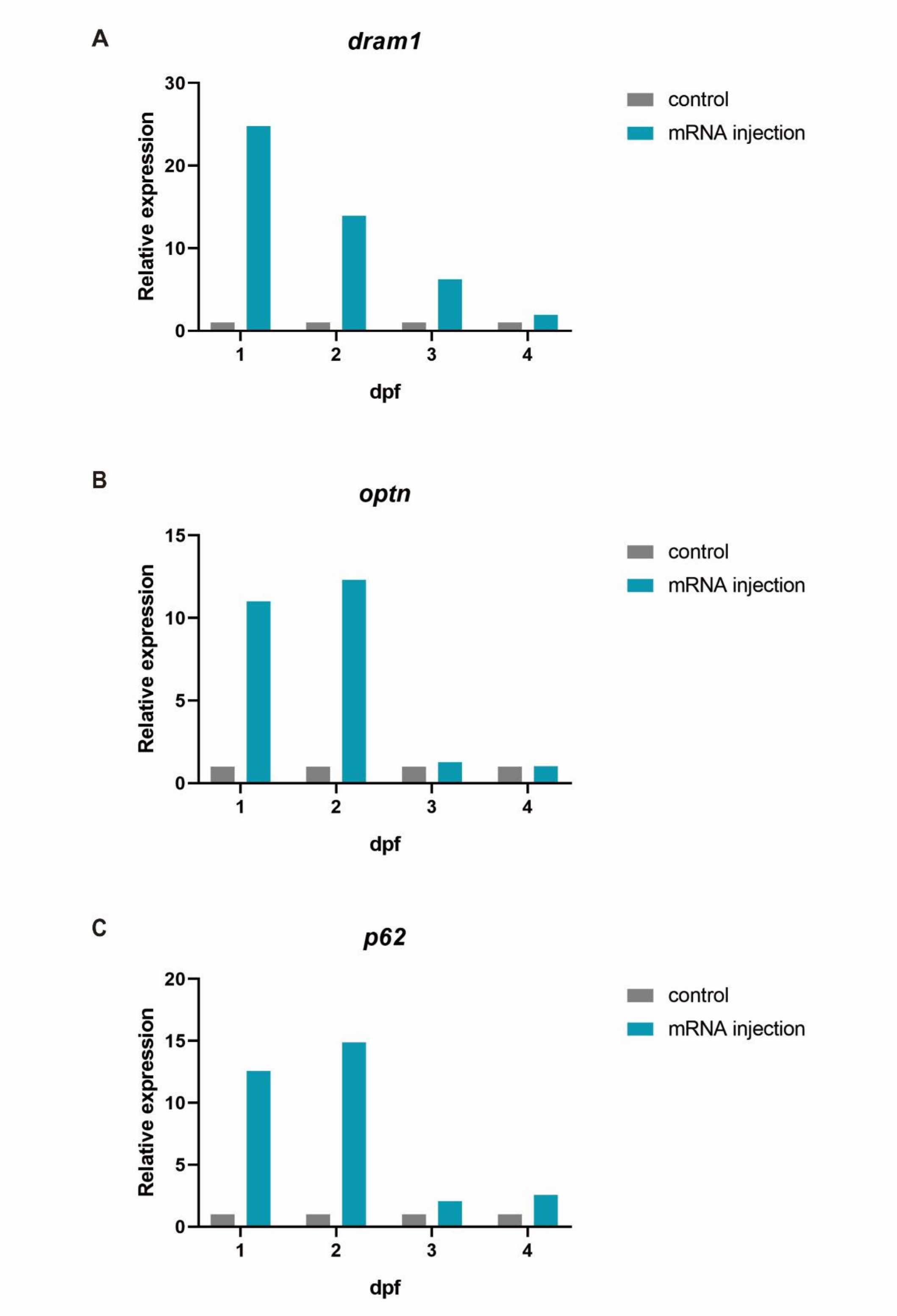

Supplementary Figure 1: Relative expression levels of *dram1*, *optn* and *p62* after injecting mRNA in embryos. A-C. *dram1, optn and p62* mRNAs were synthesized in vitro and injected into embryos at the one cell stage. Samples were collected at 1-4 dpf for qPCR measurement. Data are displayed relative to the mock injection group.

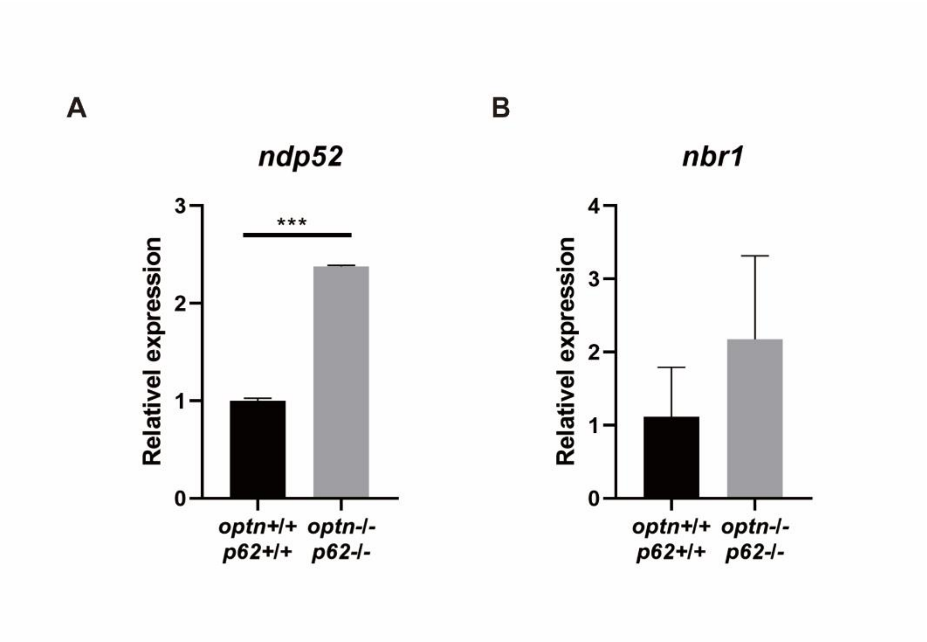

Supplementary Figure 2: Expression levels of *ndp52* (A) and *nbr1* (B) in *optn*/*p62* double mutant and wildtype larvae after Mm infection. qPCR analysis was performed at 2 dpi on RNA isolated from pools of 15 embryos infected with 200 CFU of Mm according to the indicated workflow, performed in two replicates. Data are displayed relative to the expression level in wildtype embryos, set at 1. Expression of *ndp52* was significantly higher in *optn*/*p62* mutants compared to the wildtype. ***p<0.001.
